# Predictive value of the KELIM in neoadjuvant treatment for patients with advanced ovarian cancer

**DOI:** 10.3389/fonc.2025.1677070

**Published:** 2026-01-12

**Authors:** Yicong Wang, Yongai Yu, He Yuan, Lei Wang, Pengfei Bian, Yiming Yang, Jiao Wang

**Affiliations:** Department of Gynecology and Obstetrics, Central Hospital of Dalian University of Technology, Dalian Municipal Central Hospital, Dalian, China

**Keywords:** KELIM, advanced ovarian cancer, neoadjuvant chemotherapy, interval debulking surgery, chemosensitivity

## Abstract

**Objective:**

To explore the significance of modeled CA-125 elimination rate constant (KELIM) in predicting satisfactory cytoreduction at interval debulking surgery (IDS), survival prognosis, and platinum-based chemosensitivity in patients with advanced ovarian cancer treated with neoadjuvant chemotherapy followed by interval debulking surgery (NACT-IDS).

**Methods:**

The clinical information and follow-up data of 70 patients with advanced ovarian cancer who underwent NACT-IDS in the Department of Gynecology of Dalian Central Hospital from January 2010 to June 2023 were retrospectively analyzed. The KELIM for each patient during neoadjuvant chemotherapy was calculated using the online calculation tool (https://www.biomarker-kinetics.org/CA-125-neo), and its predictive value for IDS surgical outcome, prognosis, and platinum-resistant recurrence (PRR) was analyzed.

**Results:**

A total of 70 patients met the inclusion criteria. The median follow-up time was 32 (range 6–116) months. KELIM was an independent factor for predicting satisfactory debulking at IDS. Patients with higher KELIM had a higher probability of achieving satisfactory debulking (1.40 vs. 0.61, P < 0.05). KELIM and IDS surgical outcomes were independent influencing factors for progression-free survival (PFS) and overall survival (OS). The median PFS and OS in patients with KELIM ≥1 were significantly higher than those in patients with KELIM <1 (26 months vs. 18 months, P < 0.05; 39 vs. 28 months, P < 0.05). KELIM ≥1 is an independent protective factor for subsequent recurrence of platinum resistance in patients with NACT-IDS. The median KELIM value of the platinum-sensitive recurrent group was significantly higher than that of the PRR group (1.30 vs. 0.73, P < 0.05). For patients with high KELIM, the risk of PRR is low even if IDS cytoreductive surgery is not satisfactory.

**Conclusions:**

KELIM is an important parameter to consider when performing IDS. The KELIM and IDS outcomes are independent predictors of the prognosis and PRR risk of patients with NACT-IDS. Even if cytoreduction is unsatisfactory, patients with high KELIM still have a lower risk of subsequent recurrence of platinum resistance.

## 1 Background

Ovarian cancer is the third most common gynecologic malignancy in the world and the most lethal gynecologic malignancy. The standard first-line treatment involves primary debulking surgery followed by platinum-based chemotherapy. Studies have reported that achieving satisfactory cytoreduction is an important factor affecting the survival and prognosis of patients with ovarian cancer (1–4). However, patients with advanced ovarian cancer frequently present with multiorgan metastases, and satisfactory cytoreduction is difficult to achieve. The use of NACT-IDS can reduce surgical blood loss and perioperative complications and increase the probability of satisfactory cytoreduction. The National Comprehensive Cancer Network guidelines recommend that patients who respond to chemotherapy or whose condition is stable should undergo IDS after three to four cycles of neoadjuvant chemotherapy, with decisions tailored to individual patient conditions (5). Therefore, predicting the sensitivity of patients with neoadjuvant chemotherapy(NACT) to chemotherapy is an important part of individualized therapy, informing the timing of IDS intervention or adjusting chemotherapy regimens when needed.

In 2013, You et al. developed the modeled CA125 elimination rate constant K (KELIM) as a chemotherapy sensitivity marker. Recently, the application of KELIM in ovarian cancer has gained significant research interest. KELIM can predict the likelihood of achieving satisfactory cytoreduction and has a certain correlation with platinum-resistant relapse, progression-free survival (PFS), and overall survival (OS) of patients. This study investigates patients with ovarian cancer receiving neoadjuvant chemotherapy combined with IDS to examine the relationship between KELIM and the satisfaction of intermediate tumor cell reduction, survival outcomes, and platinum sensitivity, while evaluating its predictive potential.

## 2 Methods

This retrospective study analyzed 70 patients with advanced epithelial ovarian cancer (EOC) who were treated in the Department of Gynecology at Dalian Central Hospital between January 2010 and June 2023. Eligible patients were aged 18 years or older and had International Federation of Gynecology and Obstetrics (FIGO) stage III–IV disease, including histologic subtypes such as serous, endometrioid, clear cell, and mucinous carcinoma.

Assignment to the neoadjuvant chemotherapy (NACT) group was based on a high predicted risk of suboptimal primary cytoreduction, defined as a Suidan score≥ 3 or a Fagotti score≥ 8. Surgical fitness was assessed using the Eastern Cooperative Oncology Group (ECOG) performance status and the American Society of Anesthesiologists (ASA) classification. FIGO staging was determined through a combination of imaging, including gynecological ultrasound, contrast-enhanced computed tomography (CT), magnetic resonance imaging (MRI), and/or positron emission tomography-CT (PET-CT) and cytological or histopathological confirmation via ascitic fluid analysis or laparoscopic biopsy. All serous carcinomas were confirmed as high-grade serous carcinoma (HGSC) on histologic evaluation; no cases of low-grade serous carcinoma (LGSC) were included. Tumor differentiation (low, moderate, or high grade) was documented as a pathological characteristic within the confirmed subtypes.

Treatment consisted of two to four cycles of platinum-based NACT, followed by interval debulking surgery (IDS) and subsequent adjuvant chemotherapy. All patients received chemotherapy administered every three weeks. The core regimen remained consistent throughout treatment, with the only modification being the addition of bevacizumab during adjuvant therapy for a subset of high-risk patients after IDS. After completing primary treatment, patients were followed every 2–4 months for the first 2 years, every 3–6 months for years 3-5, and annually thereafter. Follow-up visits included assessment of clinical symptoms, serum CA-125 testing, gynecological examination, and imaging (gynecological ultrasound or whole-abdomen CT). The final follow-up date was June 30, 2023, which served as the data cut-off for analysis. Progression-free survival (PFS) was defined as the time from diagnosis to the first documented disease progression or death from any cause. Patients without an event were censored on the date of their last disease-free assessment. Overall survival (OS) was calculated from diagnosis to death from any cause; surviving patients were censored on the cut-off date.

Exclusion criteria comprised non-EOC histology, metastatic ovarian cancer, prior primary cytoreductive surgery, chemotherapy-only treatment, or incomplete clinical data.

Data collected included age at diagnosis, body mass index, Eastern Cooperative Oncology Group performance status, comorbidities, FIGO stage, tumor grade, histological type, surgical outcome, surgical complications, baseline CA125 level and measurement date, CA125 level and measurement date before NACT, CA125 level and measurement date before IDS, NACT date, number of NACT cycles, NACT chemotherapy regimen, platinum-free interval (PFI), PFS, and OS. The surgical outcome was assessed according to the 2009 Gynecologic Oncology Group (GOG) criteria. Satisfactory cytoreduction was defined as no macroscopic residual disease (R0) or minimal residual disease with the largest nodule ≤1 cm (R1). Unsatisfactory cytoreduction was defined as gross residual disease with any nodule >1 cm (R2). Patients were subsequently grouped into satisfactory (R0/R1) and unsatisfactory (R2) cohorts for analysis. Importantly, R2 resection indicated surgically challenging disease in chemotherapy-responsive patients, not progression, and subsequent growth was therefore analyzed as recurrence. Platinum-free interval (PFI) was defined as the time from the last dose of postoperative platinum-based chemotherapy to the first documented disease recurrence or progression. Early relapse or platinum-resistant recurrence (PRR) was specifically defined as recurrence within 6 months from the end of postoperative chemotherapy.

### 2.1 Calculation of the KELIM score

The KELIM score was calculated using an online tool developed by You et al. at https://www.biomarker-kinetics.org/CA-125-neo, by entering the date of each neoadjuvant chemotherapy cycle and CA125 levels in the 100 days before neoadjuvant chemotherapy for each patient, including at least the values before cycles 2, 3, and 4. If <3 values are present within the first 100 days of neoadjuvant chemotherapy, the baseline CA125 is used. Based on the KELIM, patients were categorized into a favorable group (KELIM ≥1) and an unfavorable group (KELIM <1).

### 2.2 Statistical analysis

IBM SPSS Statistics version 29.0 was used for statistical analysis of the data, and the chi-square test was used for between-group comparison. Logistic regression analysis was performed to screen independent factors affecting the satisfactory cytoreduction at IDS and the subsequent recurrence of platinum resistance. Independent factors affecting PFS and OS were screened by Cox regression analysis, survival curves were estimated by the Kaplan–Meier method, and survival differences between groups were compared by the log-rank test. R software version 4.3.2 was used to plot the probability curves for predicting IDS satisfactory cytoreduction and PRR. A two-sided P value < 0.05 was considered statistically significant.

## 3 Results

A total of 70 patients with advanced ovarian cancer meeting the inclusion criteria were included in this study. The median follow-up was 32 (range 6–116) months. Demographic parameters are described in **Table 1**. The cohort included 67 cases (95.7%) of serous carcinoma and 3 cases (4.3%) of non-serous carcinoma, comprising 2 clear cell carcinomas and 1 mucinous carcinoma. Satisfactory cytoreduction (R0/R1) was achieved in 37 cases (52.9%). The main neoadjuvant chemotherapy regimen was paclitaxel combined with platinum, and the PFI of <6 months and ≥6 months after the first-line treatment accounted for 50% of the patients. The cohort included 33 patients (47.1%) with KELIM ≥1 and 37 patients (52.9%) with KELIM <1. The median CA125 before NACT was 1435.50 (734.60–3482.59) U/ml, and the median CA125 before IDS was 167.02 (66.50–474.85) U/ml.

**Table 1:** Baseline demographics and clinical characteristics.

Characteristic	N = 70
Age, years	
Median	61
Range	33–83
Body mass index (kg/m²)	
Median	24
Range	18–35
ECOG performance status, no. (%)	
0	28 (40)
1	37 (53)
2	5 (7)
3	0 (0)
Complication, no. (%)	
Hypertension	26 (37)
Diabetes	5 (7)
Coronary heart disease	5 (7)
Brain infarction	2 (3)
FIGO stage, no. (%)	
III	64 (91)
IV	6 (9)
Pathological type, no. (%)	
Serous	67 (96)
Nonserous	3 (4)
Degrees of differentiation, no. (%)	
Low	66 (95)
Moderate	3 (4)
High	1 (1)
IDS outcome, no. (%)	
R0/R1	37 (53)
R2	33 (47)
Preoperative NACT course, no. (%)	
<3	52 (74)
3	17 (24)
4	1 (2)
Total Cycles (median, IQR)	6 (5-6)
Chemotherapy regimens, no. (%)	
Paclitaxel + carboplatin	31 (44)
Paclitaxel + cisplatin	28 (40)
Paclitaxel + lobaplatin	4 (6)
Nab-paclitaxel + carboplatin	7 (10)
Platinum-free treatment interval, no. (%)	
<6 months	35 (50)
≥6 months	35 (50)
KELIM, no. (%)	
≥1	33 (47)
<1	37 (53)

### 3.1 Predictive evaluation of the KELIM on satisfactory IDS

**Figure 1:**
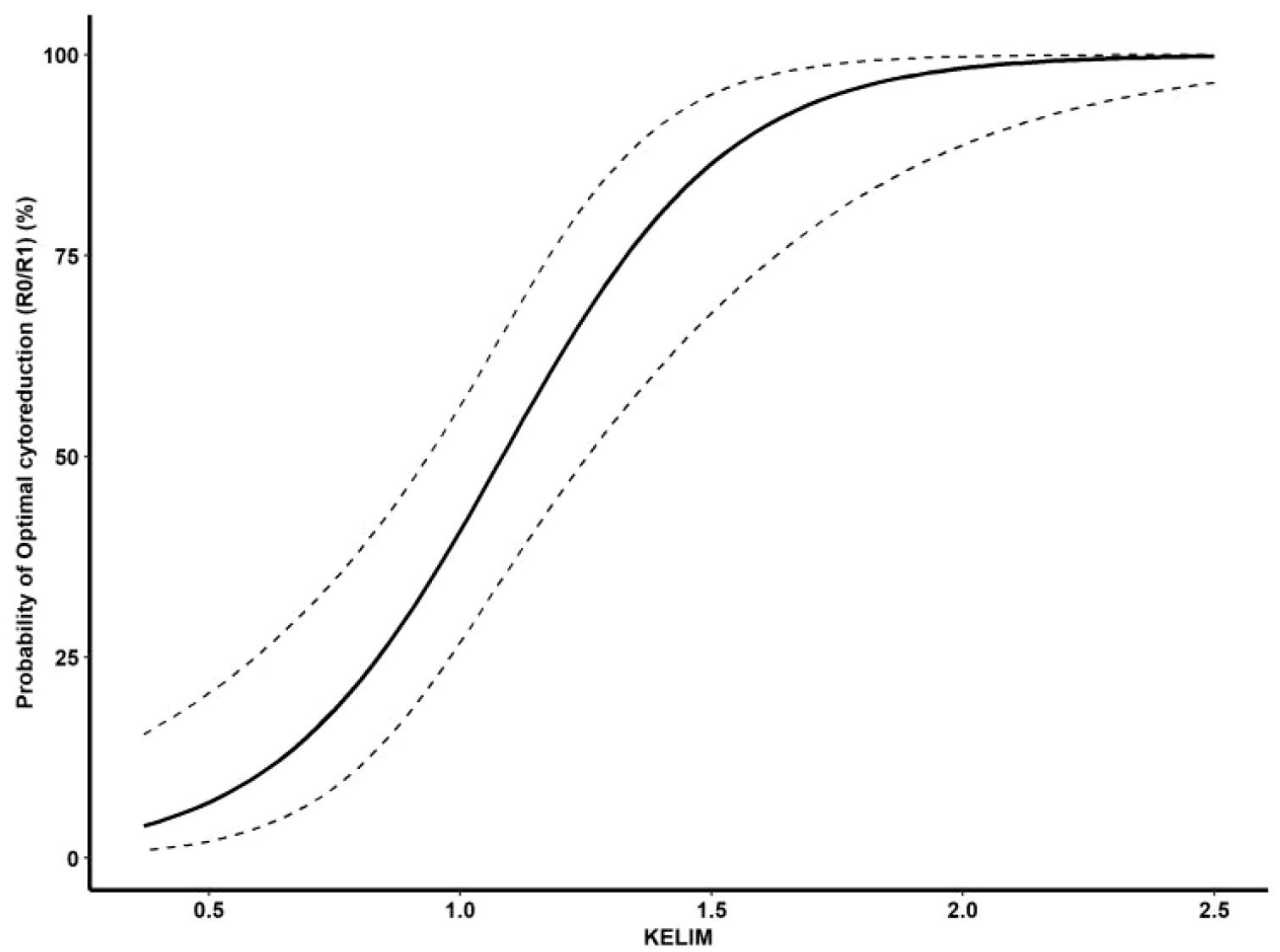
Predicted probability of satisfactory IDS according to the KELIM.

In the univariate analysis, the CA125 level before IDS (P = 0.043) and KELIM (P < 0.001) were significantly associated with satisfactory cytoreduction. In the multivariate logistic regression analysis, only KELIM (OR = 0.033, 95%CI 0.008–0.132, P < 0.001) was an independent factor in predicting satisfactory cytoreduction (**Table 2**, **Supplementary Table S1**). R software V 4.3.2 was used to generate a prediction curve for satisfactory cytoreduction at IDS based on KELIM (**Figure 1**).

**Table 2:** Analysis of factors affecting the outcome of IDS.

	Univariate factor analysis			Multifactor analysis			
	OR 95%CI P			OR 95%CI P			
CA125 Before IDS (U/ml)	1.001	1.000–1.002	0.043				
KELIM			<0.001			<0.001	
<1	REF	REF		REF	REF		
≥1	0.025	0.006–0.093		0.033	0.008–0.132		

REF, reference.

### 3.2 Predictive value of KELIM for PFS and OS

**Figure 2:**
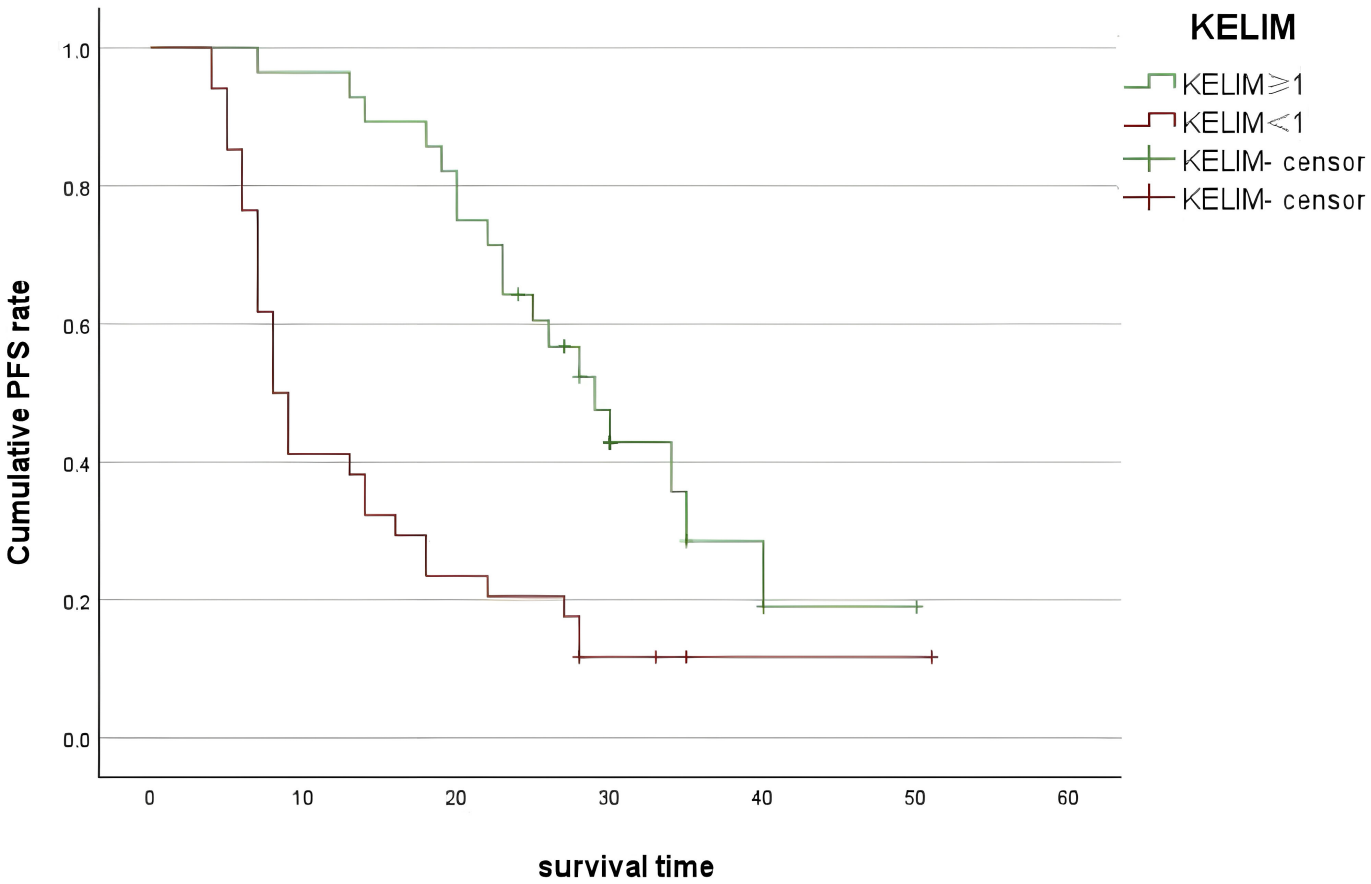
Progression-free survival curves for patients with KELIM of ≥1 and KELIM of <1..

The median PFS of all patients was 20 (14–25) months. Multivariable Cox regression analysis results showed that the KELIM (OR = 0.519, 95% CI 0.277–0.973, P = 0.041) and unsatisfactory IDS (OR = 1.911, 95% CI 1.018–3.582, P = 0.041) were the independent influencing factors of PFS (**Supplementary Table S2**). In terms of PFS, KELIM < 1 was associated with a significantly lower median PFS compared to KELIM ≥1 (18 vs. 26 months; P = 0.009) (**Figure 2**).

**Figure 3:**
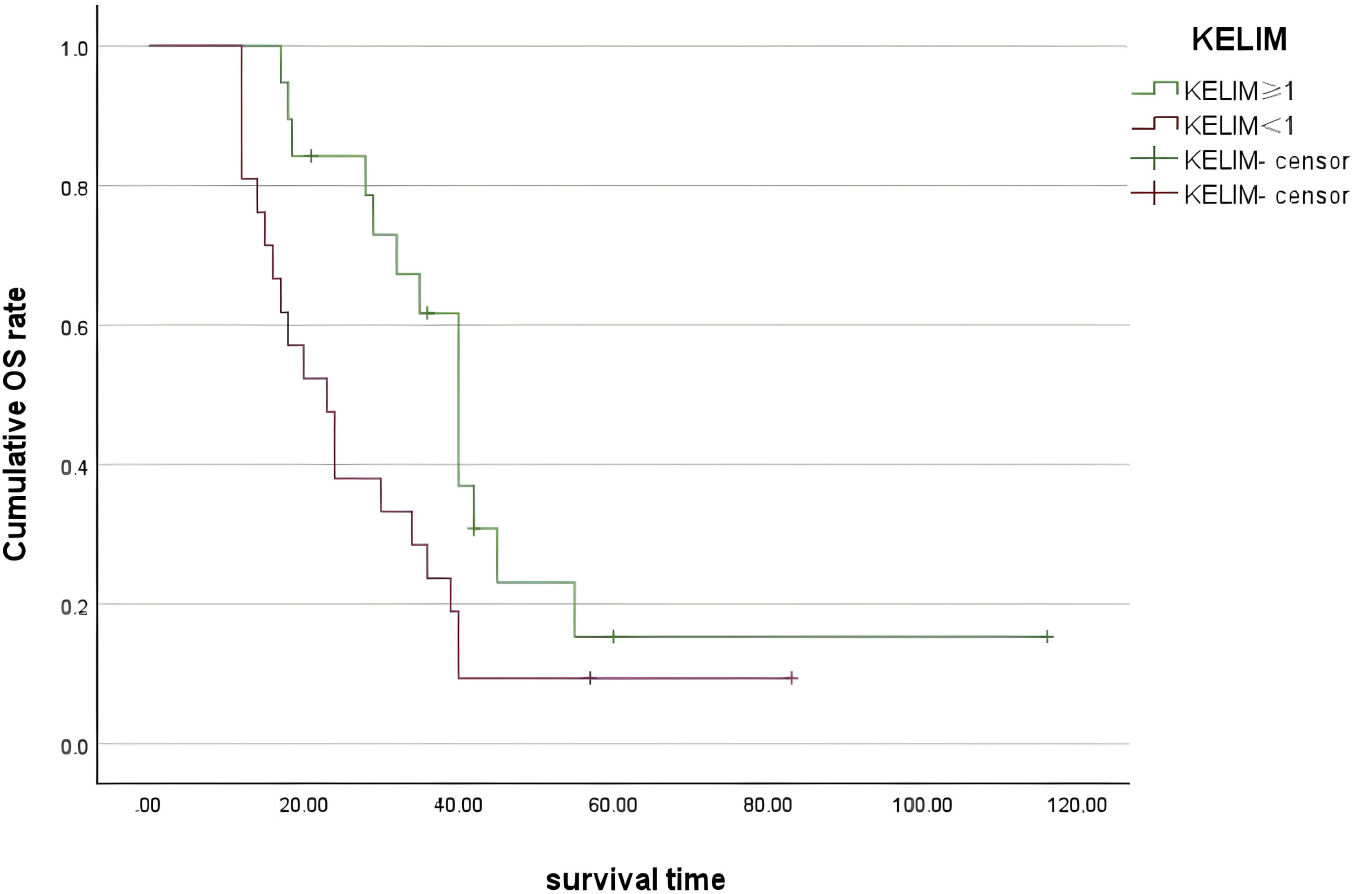
OS survival curves for patients with KELIM of ≥1 and KELIM of <1.

The median OS of all patients was 31 (25–39) months. Multivariate Cox regression analysis showed that KELIM (OR = 0.401, 95% CI 0.317–1.665, P = 0.014) and IDS surgical outcome (OR = 2.367, 95% CI 1.024–5.475, P = 0.044) were the independent influencing factors of OS in NACT-IDS patients (**Supplementary Table S3**). Patients with KELIM <1 had a significantly lower median OS than those with KELIM ≥1 (28 vs. 39 months, P = 0.003) (**Figure 3**).

### 3.3 Predictive value of KELIM for PFI

**Figure 4:**
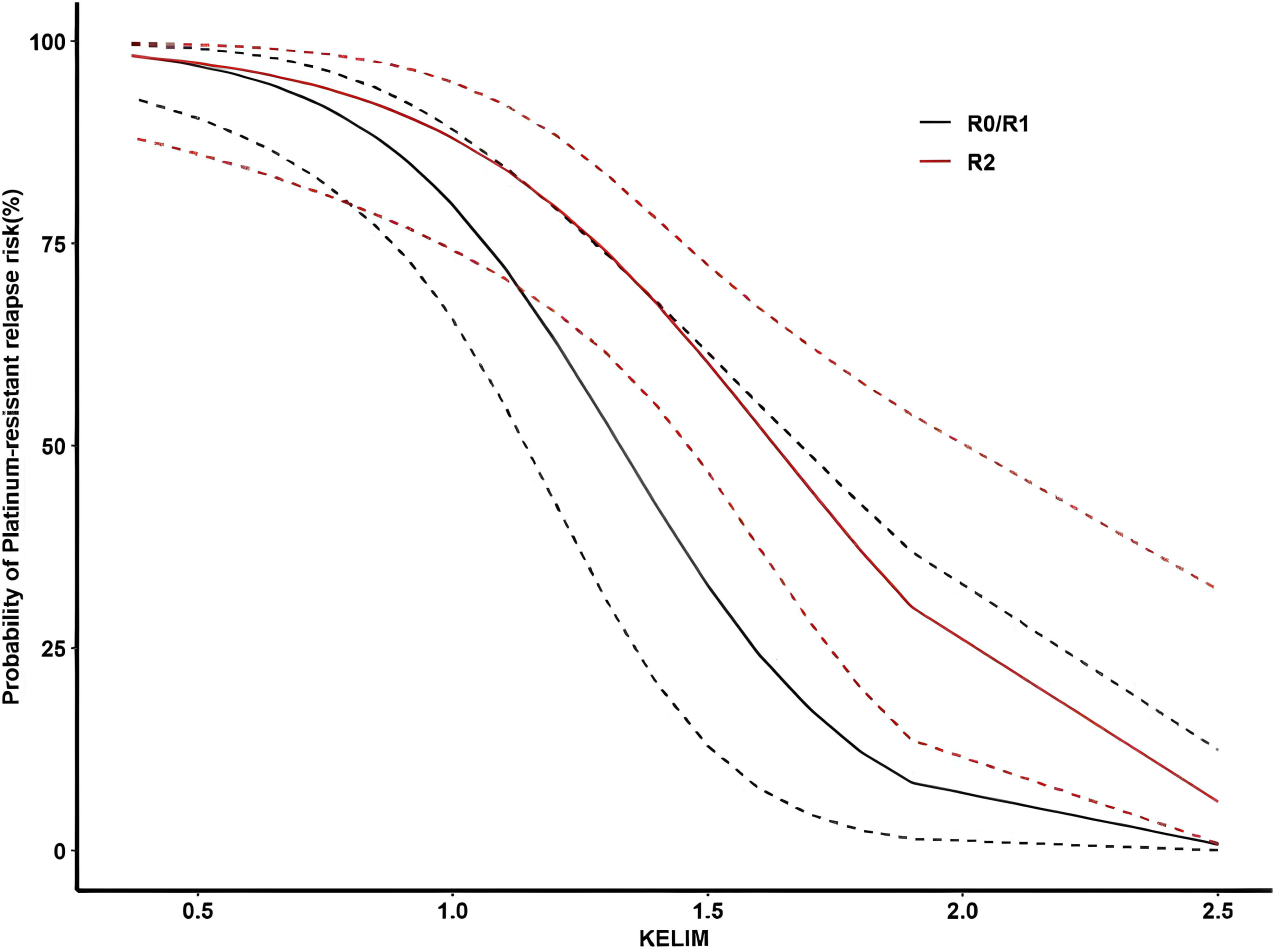
Prediction of the probability of platinum-resistant recurrence risk according to the KELIM and IDS outcomes. Black curve: recurrence probability curve of platinum resistance in patients with satisfactory IDS reduction (R0/R1). Red curve: recurrence probability curve of platinum resistance in patients with IDS unsatisfactory reduction (R2).

In this study, 50% (35/70) of patients relapsed within 6 months after the first-line treatment, which was defined as PRR. Multivariate logistic regression analysis showed that KELIM ≥1 (OR = 0.030, 95% CI 0.005–0.182, P < 0.001) was an independent protective factor and unsatisfactory cytoreduction (OR = 9.048, 95% CI 1.619–50.550, P = 0.012) was an independent risk factor for predicting the subsequent recurrence of platinum resistance (**Supplementary Table S4**, **Supplementary Table S5**). R software V.4.3.2 was used to generate a prediction curve for PRR probability based on KELIM and IDS outcomes (**Figure 4**).

**Figure 5:**
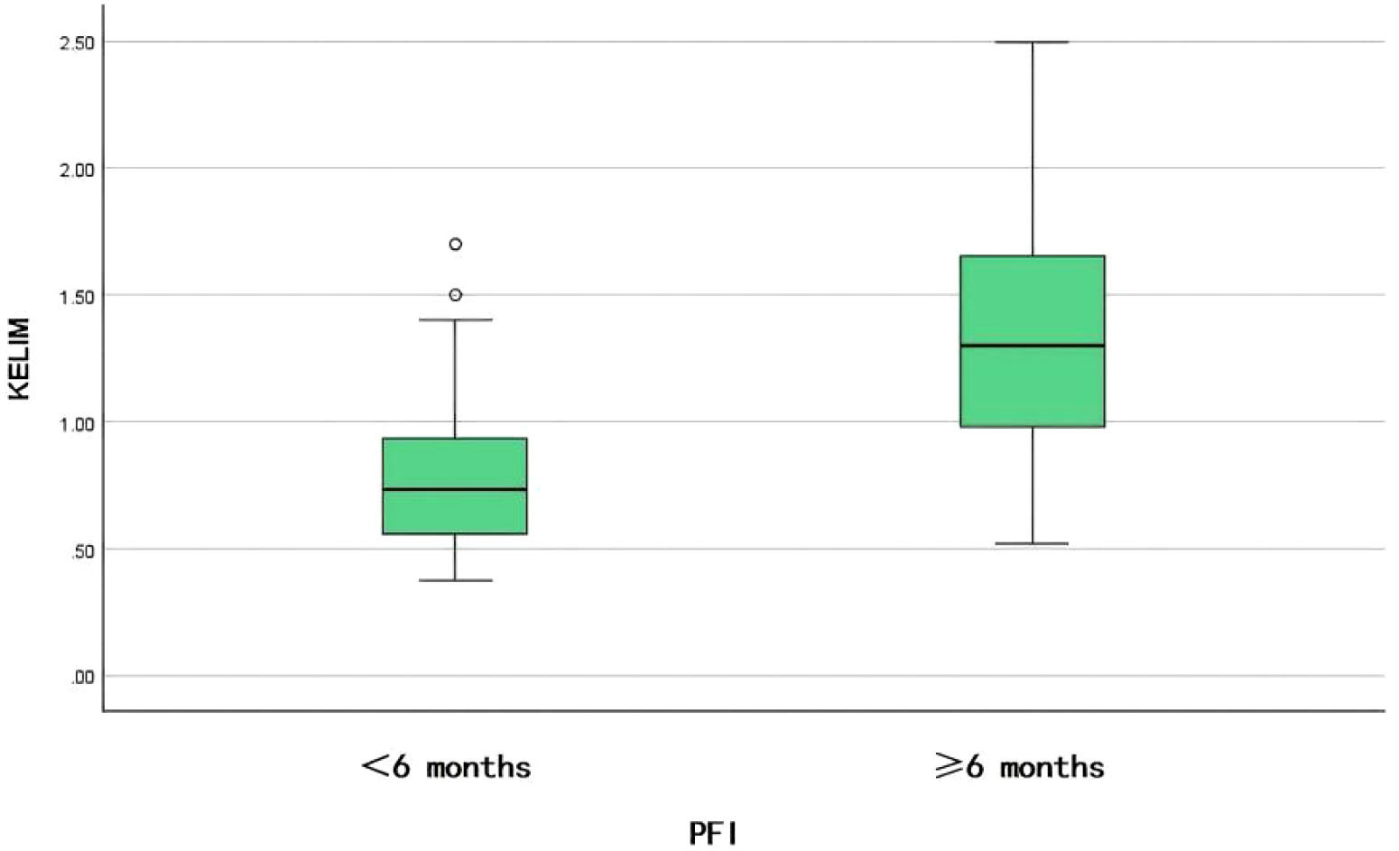
Comparison of the KELIM score between the PRR and PSR.

About 50% (35/70) of patients had PRR, and the median KELIM was 0.73 (95% CI 0.59–0.86). The other 50% (35/70) of patients had platinum-sensitive recurrence (PSR), and the median KELIM was 1.30 (95% CI 1.0–1.5). The difference between the two groups was statistically significant (Z = 4.083, P < 0.001) (**Figure 5**).

## 4 Discussion

The first-line treatment modality for patients with advanced EOC remains surgery, followed by postoperative platinum-based adjuvant chemotherapy. Patients with advanced ovarian cancer often present with multiple organ metastases, and initial satisfactory cytoreductive surgery is difficult to achieve. Several randomized clinical trials have reported that patients who are not suitable for initial cytoreductive surgery can undergo interval surgery after neoadjuvant chemotherapy, and both approaches yield comparable survival outcomes (6–9). The importance of thoroughness of cytoreductive surgery for the treatment and prognosis has been well established. However, in recent years, many studies have shown that the sensitivity of primary tumor chemotherapy also has an important impact on the feasibility and likelihood of achieving optimal cytoreduction at IDS, the efficacy of first-line treatment and maintenance treatment, and the prognosis of patients. Therefore, gynecologic oncologists always experience difficulty in accurately identifying patients with primary chemotherapy resistance, in finding better predictors the success rate of cytoreductive surgery, and in selecting the most beneficial treatment for patients with advanced ovarian cancer.

### 4.1 Correlation between KELIM and satisfactory cytoreduction in IDS

The serum CA125 level is easy to measure and is often used as an indicator to monitor the tumor status in clinical practice. Its rise and fall usually reflect the occurrence, development, and regression of EOC to a certain extent. Some researchers have attempted to predict the effects of IDS by determining a threshold of CA125 (10–12). Because the CA125 threshold was set arbitrarily by different authors after comparing the predicted values of different cutoff values, these simple static methods rely on a single point in time and do not consider the intra- and interindividual differences associated with tumor biomarker detection; thereby, their reproducibility is limited (13). KELIM minimizes the impact of within-individual variation by using mathematical models to evaluate CA-125 kinetic curves for each patient individually. Zouzoulas et al. retrospectively analyzed the clinical data of 83 patients in a single center and found a statistically significant correlation between KELIM and residual lesions (p < 0.05) (14). Using data from the CHIVA trial, You et al. revealed that the median KELIM value of patients who achieved satisfactory IDS was significantly higher than that of patients who did not achieve satisfactory IDS (1.04 vs. 0.54, p < 0.01). Multivariable logistic regression analysis showed that only KELIM was significantly associated with the likelihood of achieving a satisfactory IDS (OR 16.13,95% CI 5.51–53.38, p < 0.001) (15). In the Katic study, only 23% of patients with KELIM <1 received a satisfactory IDS, whereas 80% of patients with KELIM ≥1 achieved satisfactory cytoreduction (16). The above results indicate that patients with poor KELIM often have difficulty in achieving satisfactory cytoreduction, whereas patients with good KELIM are more likely to achieve satisfactory IDS. In this study, the median KELIM value of the IDS satisfactory group was significantly higher than that of the unsatisfactory group. Multivariate logistic analysis revealed that KELIM was an independent prognostic factor for satisfactory cytoreduction, which was consistent with the results of previous studies.

In conclusion, the KELIM is a predictive tool that can safely classify patients and predict the feasibility of complete IDS after neoadjuvant chemotherapy in a noninvasive manner, thereby selecting the appropriate IDS population.

### 4.2 Correlation between KELIM and the prognosis of ovarian cancer

Postoperative residual disease is one of the most important factors affecting the prognosis of patients with ovarian cancer. Patients with ovarian cancer without gross residual disease have a higher survival rate than those with gross residual disease (17). However, a Dutch study reported that perioperative CA125 changes were more predictive of outcomes than postoperative residual cancer (18). The trial by Li et al. found that dynamic changes in serum CA125 were the most important independent prognostic factor in patients with EOC (19). The KELIM is based on the assumption that a mathematical model of CA125 decline seems to better describe CA125 changes and its prognostic role. In the CHIVA trial, patients were stratified by KELIM into favorable (≥1.0), intermediate (0.5–1.0), and unfavorable (<0.5) groups. This study confirmed the independent prognostic value of KELIM relative to other prognostic factors (15). In a validation trial of KELIM, Piedimonte et al. reported that patients with KELIM of ≥1 had a median PFS that was 6 months longer and a 5-year OS rate of 15% higher (20). A meta-analysis of 5,842 patients presented at the 2021 ESMO annual meeting, and the results also confirmed the independent prognostic value of KELIM in PFS and OS of patients with ovarian cancer (21). This study also found that patients with KELIM ≥1 had longer median PFS and OS compared to those with KELIM <1, indicating that KELIM can predict the survival prognosis of patients with EOC.

### 4.3 Predictive value of KELIM for PRR

Although most patients with ovarian cancer are sensitive to initial chemotherapy and achieve clinical complete remission with aggressive treatment with standard first-line regimens, >70% of patients with advanced disease will have disease recurrence within 2–3 years. The interval between the last platinum-based chemotherapy cycle and recurrence, namely, the PFI, can reflect the sensitivity of patients to platinum-based chemotherapy. Several studies have reported an association between KELIM and PFI and PRR. The analysis of the CHIVA trial dataset revealed that PFI was prolonged with the increase of individual KELIM, and in the multivariate logistic regression model, KELIM and satisfactory cytoreduction at IDS were independent prognostic factors for subsequent recurrence of platinum resistance (15). In addition, Bouvarel et al. found that a higher KELIM and satisfactory cytoreduction in IDS were significantly associated with a reduced risk of early recurrence (22). In the analysis of 2,868 patients in the ICON-7, AGO-OVAR 7, and AGO-OVAR 9 pooled, KELIM was significantly associated with the risk of subsequent PRR (OR 0.17, 95% CI 0.11–0.25) (23). Thus, You et al. established a PRR risk prediction model consisting of KELIM and IDS satisfaction in the CHIVA cohort, which was externally validated by Oufkir et al. This model represents an effective tool for early adjustment of treatment decisions. However, Oufkir et al. also suggested that more data and more sophisticated models are needed to better understand the interaction between clinical and biological parameters relevant to the prediction of platinum sensitivity (15, 24). In this study, multivariate logistic regression results demonstrated that KELIM ≥1 was an independent protective factor for predicting PRR in patients with NACT-IDS, and unsatisfactory cytoreduction was an independent risk factor. The prediction curve of the recurrence probability of platinum resistance indicated that satisfactory cytoreductive surgery and higher KELIM were important factors in reducing the PRR. For patients with high KELIM, even if IDS cytoreductive surgery was unsatisfactory, the risk of PRR was still low.

This study has certain limitations inherent to its retrospective design. The analysis was limited by the absence of quantitative tumor burden metrics, such as the Peritoneal Carcinomatosis Index, and standardized surgical complexity scores, which were not routinely available in the clinical records; future studies incorporating these measures could further elucidate the relationship between chemosensitivity and the surgical effort required for optimal cytoreduction. The study was also unable to perform a comparative analysis between KELIM and RECIST 1.1 or CA125 thresholds due to a lack of systematic data. Furthermore, the sample size was limited, and some patients were excluded due to having fewer than three CA125 values during the study period. Finally, the subgroup of patients receiving first-line maintenance therapy was too small, and the treatment duration too short, to allow for meaningful further analysis.

In conclusion, KELIM was found to be an important parameter to consider when performing IDS. The KELIM and IDS outcomes are independent predictors of prognosis and PRR risk of patients with NACT-IDS. Even if cytoreduction is unsatisfactory, patients with high KELIM still have a lower risk of subsequent PRR. At present, most studies on KELIM are retrospective studies and *post hoc* analyses of clinical trials. Prospective studies are still warranted to evaluate KELIM so that it can be used more widely and comprehensively in clinical practice.

## Data Availability

The original contributions presented in the study are included in the article/**Supplementary Material**. Further inquiries can be directed to the corresponding author.

## Supplementary material

Supplementary Table 1 Univariate and multivariate analyses for predicting satisfactory cytoreduction in IDS. Abbreviation: REF, reference.

Supplementary Table 2 Analysis of factors affecting PFS in patients with NACT-IDS. Abbreviation: REF, reference.

Supplementary Table 3 Analysis of factors affecting OS in patients with NACT-IDS. Abbreviation: REF, reference.

Supplementary Table 4 Univariate and multivariate analyses for predicting the subsequent recurrence of platinum resistance in patients with NACT-IDS. Abbreviation: REF, reference.

Supplementary Table 5 Univariate and multivariate analyses affecting the recurrence of platinum resistance after NACT-IDS. Abbreviation: REF, reference.
