# Supplementary material for: Predictive value of the KELIM in neoadjuvant treatment for patients with advanced ovarian cancer
Source: Front Oncol. 2026 Jan 12;15:1677070. doi: 10.3389/fonc.2025.1677070 (PMC12832227; doi:10.3389/fonc.2025.1677070)
Supplement: Supplementary Table 1 — Univariate and multivariate analyses for predicting satisfactory cytoreduction in IDS. Abbreviation: REF, reference. [file DataSheet1.docx]

Supplementary Table S1 Univariate and multivariate analyses for predicting satisfactory cytoreduction in IDS

|  | Univariate factor analysis | | |  | Multi-factor analysis | | |
| --- | --- | --- | --- | --- | --- | --- | --- |
|  | OR | 95%CI | *P* |  | OR | 95%CI | *P* |
| Age, years | 1.023 | 0.976-1.072 | 0.341 |  |  |  |  |
| BMI(kg/m²) | 0.934 | 0.815-1.070 | 0.326 |  |  |  |  |
| ECOG |  |  |  |  |  |  |  |
| 0 | REF | REF |  |  |  |  |  |
| 1 | 1.176 | 0.440-3.145 | 0.746 |  |  |  |  |
| 2&3 | 1.5 | 0.216-10.402 | 0.682 |  |  |  |  |
| Complication |  |  | 0.088 |  |  |  |  |
| yes | REF | REF |  |  |  |  |  |
| no | 0.401 | 0.140-1.146 |  |  |  |  |  |
| FIGO stage |  |  | 0.152 |  |  |  |  |
| III | REF | REF |  |  |  |  |  |
| IV | 5 | 0.553-45.226 |  |  |  |  |  |
| Pathological type |  |  | 0.379 |  |  |  |  |
| serous | REF | REF |  |  |  |  |  |
| Non-serous | 2.824 | 0.279-28.562 |  |  |  |  |  |
| Degrees of differentiation |  |  | 0.311 |  |  |  |  |
| low | REF | REF |  |  |  |  |  |
| moderate&high | 0.413 | 0.074-2.289 |  |  |  |  |  |
| Course of Preoperative NACT |  |  | 0.224 |  |  |  |  |
| ＜3 | REF | REF |  |  |  |  |  |
| ≥3 | 2.824 | 0.531-15.022 |  |  |  |  |  |
| Course of chemotherapy |  |  | 0.945 |  |  |  |  |
| Paclitaxel + carboplatin | REF | REF |  |  |  |  |  |
| others | 0.967 | 0.375-2.497 |  |  |  |  |  |
| CA125 before NACT(U/mL) | 1.435 | 1.117-1.532 | 0.88 |  |  |  |  |
| CA125 before IDS(U/mL) | 1.001 | 1.000-1.002 | 0.043 |  |  |  |  |
| KELIM |  |  | ＜0.001 |  |  |  | ＜0.001 |
| ＜1 | REF | REF |  |  | REF | REF |  |
| ≥1 | 0.025 | 0.006-0.093 |  |  | 0.033 | 0.008-0.132 |  |

Abbreviation: REF, reference.
