# Supplementary material for: Predictive value of the KELIM in neoadjuvant treatment for patients with advanced ovarian cancer
Source: Front Oncol. 2026 Jan 12;15:1677070. doi: 10.3389/fonc.2025.1677070 (PMC12832227; doi:10.3389/fonc.2025.1677070)
Supplement: Supplementary Table 2 — Analysis of factors affecting PFS in patients with NACT-IDS. Abbreviation: REF, reference. [file DataSheet2.docx]

Supplementary Table S2 Analysis of factors affecting PFS in patients with NACT-IDS

|  | Univariate factor analysis | | |  | Multi-factor analysis | | |
| --- | --- | --- | --- | --- | --- | --- | --- |
|  | OR | 95%CI | *P* |  | OR | 95%CI | *P* |
| Age, years |  |  | 0.849 |  |  |  |  |
| ＜59 | REF | REF |  |  |  |  |  |
| ≥59 | 0.957 | 0.371-1.163 |  |  |  |  |  |
| BMI（kg/m²） |  |  | 0.106 |  |  |  |  |
| ＜24 | REF | REF |  |  |  |  |  |
| ≥24 | 0.635 | 0.366-1.102 |  |  |  |  |  |
| ECOG |  |  | 0.135 |  |  |  |  |
| 0 | REF | REF |  |  |  |  |  |
| 1 | 0.927 | 0.527-1.631 |  |  |  |  |  |
| 2&3 | 0.384 | 0.110-1.345 |  |  |  |  |  |
| complication |  |  | 0.304 |  |  |  |  |
| yes | REF | REF |  |  |  |  |  |
| no | 1.355 | 0.759-2.416 |  |  |  |  |  |
| FIGO stage |  |  | 0.519 |  |  |  |  |
| III | REF | REF |  |  |  |  |  |
| IV | 0.737 | 0.292-1.863 |  |  |  |  |  |
| pathological type |  |  | 0.263 |  |  |  |  |
| serous | REF | REF |  |  |  |  |  |
| Non-serous | 0.436 | 0.102-1.865 |  |  |  |  |  |
| degrees of differentiation |  |  | 0.855 |  |  |  |  |
| low | REF | REF |  |  |  |  |  |
| moderate&high | 0.916 | 0.356-2.356 |  |  |  |  |  |
| IDS outcome |  |  | 0.041 |  |  |  | 0.044 |
| R0/R1 | REF | REF |  |  | REF | REF |  |
| R2 | 1.525 | 1.282-1.974 |  |  | 1.911 | 1.018-3.582 |  |
| course of preoperative NACT |  |  | 0.935 |  |  |  |  |
| ＜3 | REF | REF |  |  |  |  |  |
| ≥3 | 1.027 | 0.545-1.936 |  |  |  |  |  |
| chemotherapy regimens |  |  | 0.938 |  |  |  |  |
| Paclitaxel + carboplatin | REF | REF |  |  |  |  |  |
| Others | 1.023 | 0.585-1.788 |  |  |  |  |  |
| CA125 before NACT（U/mL） |  |  | 0.287 |  |  |  |  |
| ＜1435 | REF | REF |  |  |  |  |  |
| ≥1435 | 1.346 | 0.779-2.327 |  |  |  |  |  |
| CA125 before IDS（U/mL） |  |  | 0.478 |  |  |  |  |
| ＜167 | REF | REF |  |  |  |  |  |
| ≥167 | 0.821 | 0.474-1.418 |  |  |  |  |  |
| KELIM |  |  | 0.011 |  |  |  | 0.041 |
| ＜1 | REF | REF |  |  | REF | REF |  |
| ≥1 | 0.484 | 0.276-0.846 |  |  | 0.519 | 0.277-0.973 |  |

Abbreviation: REF, reference.
