# Supplementary material for: Predictive value of the KELIM in neoadjuvant treatment for patients with advanced ovarian cancer
Source: Front Oncol. 2026 Jan 12;15:1677070. doi: 10.3389/fonc.2025.1677070 (PMC12832227; doi:10.3389/fonc.2025.1677070)
Supplement: Supplementary Table 3 — Analysis of factors affecting OS in patients with NACT-IDS. Abbreviation: REF, reference. [file DataSheet3.docx]

Supplementary Table S3 Analysis of factors affecting OS in patients with NACT-IDS

|  | Univariate factor analysis | | |  | Multi-factor analysis | | |
| --- | --- | --- | --- | --- | --- | --- | --- |
|  | OR 95%CI P | | |  | OR 95%CI P | | |
| Age, years |  |  | 0.835 |  |  |  |  |
| ＜59 | REF | REF |  |  |  |  |  |
| ≥59 | 1.076 | 0.539-2.151 |  |  |  |  |  |
| BMI（kg/m²） |  |  | 0.528 |  |  |  |  |
| ＜24 | REF | REF |  |  |  |  |  |
| ≥24 | 0.801 | 0.403-1.593 |  |  |  |  |  |
| ECOG |  |  | 0.236 |  |  |  |  |
| 0 | REF | REF |  |  |  |  |  |
| 1 | 0.478 | 0.353-1.223 |  |  |  |  |  |
| ≥2 | 0.525 | 0.452-1.523 |  |  |  |  |  |
| Complication |  |  | 0.108 |  |  |  |  |
| yes | REF | REF |  |  |  |  |  |
| no | 1.799 | 0.880-3.679 |  |  |  |  |  |
| FIGO stage |  |  | 0.465 |  |  |  |  |
| III | REF | REF |  |  |  |  |  |
| IV | 0.828 | 0.638-1.673 |  |  |  |  |  |
| Pathological type |  |  |  |  |  |  |  |
| serous | REF | REF | 0.324 |  |  |  |  |
| Non-serous | 0.723 | 0.471-1.634 |  |  |  |  |  |
| Degrees of differentiation |  |  | 0.538 |  |  |  |  |
| low | REF | REF |  |  |  |  |  |
| moderate&high | 0.813 | 0.426-1.461 |  |  |  |  |  |
| IDS outcome |  |  | 0.001 |  |  |  | 0.044 |
| R0/R1 | REF | REF |  |  | REF | REF |  |
| R2 | 3.271 | 1.582-6.763 |  |  | 2.367 | 1.024-5.475 |  |
| course of preoperative NACT |  |  | 0.878 |  |  |  |  |
| ＜3 | REF | REF |  |  |  |  |  |
| ≥3 | 1.145 | 1.003-1.515 |  |  |  |  |  |
| Chemotherapy regimens |  |  | 0.883 |  |  |  |  |
| Paclitaxel + carboplatin | REF | REF |  |  |  |  |  |
| Others | 1.053 | 0.529-2.098 |  |  |  |  |  |
| CA125 before NACT(U/mL) |  |  | 0.426 |  |  |  |  |
| ＜1435 | REF | REF |  |  |  |  |  |
| ≥1435 | 1.323 | 0.664-2.634 |  |  |  |  |  |
| CA125 before IDS(U/mL) |  |  | 0.027 |  |  |  | 0.451 |
| ＜167 | REF | REF |  |  | REF | REF |  |
| ≥167 | 0.446 | 0.218-0.912 |  |  | 0.727 | 0.317-1.665 |  |
| KELIM |  |  | 0.003 |  |  |  | 0.014 |
| ＜1 | REF | REF |  |  | REF | REF |  |
| ≥1 | 0.344 | 0.169-0.699 |  |  | 0.401 | 0.194-0.829 |  |

Abbreviation: REF, reference.
