# Supplementary material for: Predictive value of the KELIM in neoadjuvant treatment for patients with advanced ovarian cancer
Source: Front Oncol. 2026 Jan 12;15:1677070. doi: 10.3389/fonc.2025.1677070 (PMC12832227; doi:10.3389/fonc.2025.1677070)
Supplement: Supplementary Table 4 — Univariate and multivariate analyses for predicting the subsequent recurrence of platinum resistance in patients with NACT-IDS. Abbreviation: REF, reference. [file DataSheet4.docx]

Supplementary Table S4 Univariate and multivariate analyses for predicting the subsequent recurrence of platinum resistance in patients with NACT-IDS

|  | Univariate factor analysis | | |  | Multi-factor analysis | | |
| --- | --- | --- | --- | --- | --- | --- | --- |
|  | OR | 95%CI | *P* |  | OR | 95%CI | *P* |
| Age, years | 0.946 | 0.892-1.002 | 0.06 |  |  |  |  |
| BMI(kg/m²) | 0.252 | 0.782-1.067 | 0.913 |  |  |  |  |
| ECOG |  |  |  |  |  |  |  |
| 0 | REF | REF |  |  |  |  |  |
| 1 | 0.848 | 0.262-2.745 | 0.784 |  |  |  |  |
| 2&3 | 0.409 | 0.055-3.035 | 0.382 |  |  |  |  |
| Complication |  |  | 0.334 |  |  |  |  |
| yes | REF | REF |  |  |  |  |  |
| no | 0.579 | 0.191-1.754 |  |  |  |  |  |
| FIGO stage |  |  | 0.652 |  |  |  |  |
| III | REF | REF |  |  |  |  |  |
| IV | 1.667 | 0.181-15.351 |  |  |  |  |  |
| Pathological type |  |  | 0.422 |  |  |  |  |
| serous | REF | REF |  |  |  |  |  |
| Non-serous | 2.435 | 0.278-21.349 |  |  |  |  |  |
| Degrees of differentiation |  |  | 0.532 |  |  |  |  |
| low | REF | REF |  |  |  |  |  |
| moderate&high | 2.043 | 0.228-18.282 |  |  |  |  |  |
| Course of chemotherapy |  |  | 0.689 |  |  |  |  |
| ＜3 | REF | REF |  |  |  |  |  |
| ≥3 | 0.78 | 0.231-2.633 |  |  |  |  |  |
| Course of chemotherapy |  |  | 0.872 |  |  |  |  |
| Paclitaxel + carboplatin | REF | REF |  |  |  |  |  |
| others | 0.913 | 0.301-2.765 |  |  |  |  |  |
| CA125 before NACT(U/mL) | 1.001 | 1.000-1.004 | 0.119 |  |  |  |  |
| CA125 before IDS(U/mL) | 1.002 | 1.000-1.004 | 0.037 |  |  |  |  |
| KELIM |  |  | ＜0.001 |  |  |  | ＜0.001 |
| ＜1 | REF | REF |  |  | REF | REF |  |
| ≥1 | 0.03 | 0.005-0.182 |  |  | 0.018 | 0.002-0.152 |  |
| IDS outcome |  |  | 0.011 |  |  |  | 0.012 |
| R0/R1 | REF | REF |  |  | REF | REF |  |
| R2 | 4.712 | 1.422-15.622 |  |  | 9.048 | 1.619-50.550 |  |

Abbreviation: REF, reference.
