# Supplementary material for: Predictive value of the KELIM in neoadjuvant treatment for patients with advanced ovarian cancer
Source: Front Oncol. 2026 Jan 12;15:1677070. doi: 10.3389/fonc.2025.1677070 (PMC12832227; doi:10.3389/fonc.2025.1677070)
Supplement: Supplementary Table 5 — Univariate and multivariate analyses affecting the recurrence of platinum resistance after NACT-IDS. Abbreviation: REF, reference. [file DataSheet5.docx]

Supplementary Table S5 Univariate and multivariate analyses affecting the recurrence of platinum resistance after NACT-IDS

|  | Univariate factor analysis | | |  | Multi-factor analysis | | |
| --- | --- | --- | --- | --- | --- | --- | --- |
|  | OR 95%CI P | | |  | OR 95%CI P | | |
| CA125 before IDS surgery(U/ml) | 1.002 | 1.000-1.004 | 0.037 |  |  |  |  |
| KELIM |  |  | ＜0.001 |  |  |  | ＜0.001 |
| ＜1 | REF | REF |  |  | REF | REF |  |
| ≥1 | 0.03 | 0.005-0.182 |  |  | 0.018 | 0.002-0.152 |  |
| IDS outcome |  |  | 0.011 |  |  |  | 0.012 |
| R0/R1 | REF | REF |  |  | REF | REF |  |
| R2 | 4.712 | 1.422-15.622 |  |  | 9.048 | 1.619-50.550 |  |

Abbreviation: REF, reference.
